# Using Storytelling to Address Oral Health Knowledge in American Indian and Alaska Native Communities

**DOI:** 10.5888/pcd15.170305

**Published:** 2018-05-24

**Authors:** Brenda Heaton, Christina Gebel, Andrew Crawford, Judith C. Barker, Michelle Henshaw, Raul I. Garcia, Christine Riedy, Maureen A. Wimsatt

**Affiliations:** 1 Department of Health Policy and Health Services Research, Henry M. Goldman School of Dental Medicine, Boston University, Boston, Massachusetts; 2 California Tribal Epidemiology Center, California Rural Indian Health Board, Inc, Sacramento, California; 3 Department of Oral Health Policy and Epidemiology, Harvard School of Dental Medicine, Boston, Massachusetts; 4 Center to Address Disparities in Children’s Oral Health, School of Dentistry, University of California San Francisco, San Francisco, California

## Abstract

**Introduction:**

We conducted a qualitative analysis to evaluate the acceptability of using storytelling as a way to communicate oral health messages regarding early childhood caries (ECC) prevention in the American Indian and Alaska Native (AIAN) population.

**Methods:**

A traditional story was developed and pilot tested among AIAN mothers residing in 3 tribal locations in northern California. Evaluations of the story content and acceptability followed a multistep process consisting of initial feedback from 4 key informants, a focus group of 7 AIAN mothers, and feedback from the Community Advisory Board. Upon story approval, 9 additional focus group sessions (N = 53 participants) were held with AIAN mothers following an oral telling of the story.

**Results:**

Participants reported that the story was culturally appropriate and used relatable characters. Messages about oral health were considered to be valuable. Concerns arose about the oral-only delivery of the story, story content, length, story messages that conflicted with normative community values, and the intent to target audiences. Feedback by focus group participants raised some doubts about the relevance and frequency of storytelling in AIAN communities today.

**Conclusion:**

AIAN communities value the need for oral health messaging for community members. However, the acceptability of storytelling as a method for the messaging raises concerns, because the influence of modern technology and digital communications may weaken the acceptability of the oral tradition. Careful attention must be made to the delivery mode, content, and targeting with continual iterative feedback from community members to make these messages engaging, appropriate, relatable, and inclusive.

## Introduction

American Indian and Alaska Native (AIAN) communities have a high prevalence of early childhood caries (ECC), a chronic and infectious disease of childhood that originates from bacteria that can be passed from mother to child. ECC can begin soon after primary teeth erupt and can cause problems with eating, sleeping, and learning (1). Furthermore, children with ECC are 3 times more likely to develop caries in their permanent teeth (2). The prevalence of untreated caries among AIAN children was reported to be higher than among any other racial/ethnic group in the United States (3,4), despite declines in the overall prevalence of the disorder over the last several decades (5,6).

AIAN parents face obstacles (eg, distance to treatment, lack of transportation, shortage of dental clinics, costs) (7,8) that can limit their access to professionally applied preventive services (eg, fluoride varnish) and restorative care for their children. The high prevalence of ECC, its consequences, and community-specific barriers to its prevention and treatment led us to explore new and culturally appropriate ways to promote oral health and prevent disease in AIAN communities.

Storytelling is a potential mechanism to communicate health messages among AIANs (9), including oral health messages (10). AIAN communities have a storytelling tradition (11), which facilitates knowledge transfer and communication of core values and belief systems important to the tribal community. Thus, storytelling has been leveraged for health promotion and practice in AIAN communities and in other communities where the storytelling tradition is valued (12–16). We report qualitative findings on the acceptability of a health communication intervention using storytelling as a means to communicate oral health messages related to ECC prevention. The primary objective of our qualitative analysis was to evaluate a health communication intervention that employed storytelling, specifically story content and delivery, as a potential delivery mechanism for oral health promotion messages among AIAN women.

## Methods

Our study was conducted as part of the Native Oral Health Project (NOHP), which is administered by the California Tribal Epidemiology Center within the California Rural Indian Health Board, Inc (CRIHB) and was funded by the National Institute of Dental and Craniofacial Research. The The NOHP included a set of exploratory research studies designed to inform public health research, prevention and intervention, and policy by assessing oral health knowledge, beliefs, behaviors, and barriers to oral health care among mothers in AIAN communities and the acceptability of community-engaged interventions. Existing CRIHB partnerships were leveraged for access to 3 tribal communities in northern California. Advisory board members and liaisons from each tribal community assisted in facilitating the NOHP. A community advisory board composed of 8 members from the statewide California AIAN community, including the 3 communities involved in this study, provided advice on study procedures and interpretation of findings during quarterly meetings throughout the study period.

### Story development and content

A traditional story, *Coyote and Little Man*, was developed in consultation with leading experts in the field of ECC and oral health disparities, the NOHP Community Advisory Board, and traditional storytellers. It contained 10 key parental oral health messages promoted by the American Academy of Pediatric Dentistry (17). Briefly, the story content chronicles Coyote and the “little man” in his mouth (ie, bacteria that can cause a cavity). The coyote often plays the role of a trickster in many AIAN stories told in northern California. In this particular story (Appendix), Coyote tries to trick other animals into allowing Little Man’s family members to live in their mouths to relieve the pain in his mouth.

The caries prevention story, told by AIAN traditional storytellers, was delivered to a convenience sample of AIAN women. Development of the story content and its acceptability followed a multistep process consisting of feedback from key informants, the community advisory board, and a focus group of AIAN mothers. Nine focus groups of pregnant AIAN women and AIAN mothers of children under age 6 evaluated the story and the story’s delivery. This study took place from September 2012 through February 2014. The CRIHB Institutional Review Board reviewed and approved our study protocol. All participants provided written informed consent and received compensation in the form of a gift card or oral health supplies, such as toothbrushes and toothpaste.

Key informant storytellers were recruited from a pool of names based on community advisory board recommendations and were screened by telephone to determine whether they met the following criteria: 1) self-identified as an American Indian and/or Alaska Native, 2) knew and told traditional stories, 3) had more than 2 years of storytelling experience, and 4) created their stories based on traditional knowledge or used stories passed down to them from elders. At least one of the 4 storytellers was required to be an elder, defined as a member of a tribe who was aged 65 or older and who was considered by tribal members to be knowledgeable in traditional practices and culture. Recruitment of focus group participants was done through radio public service announcements, print media, social media, and by word-of-mouth communications between tribal liaisons and community organizations. Events were held at tribal facilities and resource centers in each of the 3 communities during which AIAN mothers were invited to have lunch, receive oral health materials (eg, toothbrushes, fluoridated toothpaste, dental floss), learn more about the project, and sign up for the study if interested. Women were eligible to participate if they self-identified as American Indian or Alaska Native (ie, could report tribal affiliations beyond the 3 sites in the study), were aged 18 or older, and were either pregnant or had a child aged 6 or younger. Mothers are often perceived to be the wisdom keepers and keepers of the family’s health. Therefore, the input of mothers was considered invaluable in developing an appropriate and culturally sensitive health communication intervention. Additionally, pregnancy was seen as an optimal time to educate women, before the birth of their child.

A copy of the story was mailed to participating storytellers at least one week before the scheduled one-on-one telephone interview. Interviews elicited feedback about the cultural appropriateness of the story, the potential value of a traditional story with modern-day messaging, the appropriateness of the animal characters in the story, women’s perceptions of the community’s attitude toward Coyote as a trickster, story strengths and weaknesses, and ways the story could be improved.

### Focus groups

Nine focus groups were conducted among a convenience sample of AIAN women with tribal affiliations within and outside 3 northern California tribal communities. Three focus groups were conducted in each of the 3 communities immediately following a 30-minute telling of the story by a single storyteller who was not otherwise involved in the study (ie, not a key informant). A trained facilitator, who was also a member of an AIAN community, moderated discussions by following a written guide. Discussions were audiotaped and later transcribed. Guides were developed with the assistance of qualitative researchers who received feedback and review from community advisory board members. Participants (N = 53) additionally responded in writing to open-ended questions that elicited their opinions about the storytelling session and their intention to share the story with others.

A single, qualitative data analyst who was independent of the research implementation team coded all interview and focus group transcripts by using NVivo 10 (QSR International Pty Ltd) software. Initial codes were broad in scope and assigned by using inductive coding methods (18,19) and were entered into a master list. Some initial codes were identified a priori*,* were derived from existing concepts in the literature, or were inherent in the story itself (eg, character, message) (10–16). Other codes emerged from the interview transcript (eg, relevance to tradition). Whether a priori or emergent, each initial code was reviewed or discussed with 4 members of the study team, and new refined codes or subcodes were developed, defined, and added to the master list. Overall, the master list included 18 codes and 26 subcodes. Major themes were collectively decided on by members of the study team. Thematic analysis continued iteratively, with ongoing review by members of the study team, until no new meaningful subcodes were discernible. Written feedback was similarly analyzed, though it provided no new themes or subthemes.

## Results

Four key informant storytellers were interviewed, 3 of whom were women and 3 of whom were recognized as elders in their respective communities. Five storytelling sessions were held. In addition to the pilot focus group of 7 women, a total of 53 women attended a storytelling session and subsequently participated in 9 focus groups. All participants also provided additional written feedback. Focus groups ranged in size from 3 to 8 participants (average, 6 participants). Participants were aged 18 to 51 (median age, 29 y), and approximately half reported their oral health as either fair or poor, had an annual household income at or below the federal poverty level for a household of 2 (≤$20,000), and were unemployed (Table 1). Three major themes were identified by using qualitative analysis: 1) cultural and traditional relevance of storytelling, 2) critiques of characters and messages, and 3) critiques of health messaging and targeting. Selected quotations for each major theme and related subthemes are presented in Table 2.

**Table 1:** Sociodemographic Characteristics of American Indian and Alaska Native Women (N = 53) From Three Tribal Communities Participating in Focus Groups for a Storytelling Intervention Addressing Oral Health, Northern California, September 2012–February 2014

Characteristic	N^a^
**Education **	
≤High school diploma or general equivalency degree	21
Some college	31
**Employment**	
Employed full-time or part-time	21
Unemployed (includes students, homemakers, the disabled)	29
Annual household income ≤$20,000	25
**Relationship status**	
Married or living with partner	31
Divorced, separated, or never married	19
**Age of participating mothers, y**	
18–23	13
24–29	17
30–34	11
35–51	11
**Mother reported fair or poor overall oral health for**:	
Self	28
Child	10
**Mother reported dental insurance available for:**	
Self	45
Child	45
Mother’s last dental visit was within the past 12 months	37
**Time since child's last routine checkup or cleaning**	
Within past year	36
More than 1 year ago but less than 2 years ago	6
Mother reported a time in prior year when child needed dental care, but could not get it	6

a Not all counts sum to 53 for items with multiple response categories because some participants did not respond to each item or not all response categories are shown.

**Table 2:** Qualitative Themes and Selected Quotes From American Indian and Alaska Native Women Participating in a Storytelling Intervention Addressing Oral Health, Northern California, September 2012–February 2014

Themes and Subthemes	Selected Quotations from Participants^a^
**Cultural and traditional relevance of storytelling**	“I think learning our language and keeping our culture–cultural activities going, and storytelling would be part of that.” [KI–02]
Acceptable and accessible format	**“**But when it’s a story and you’re listening to it, you’re enjoying it more, you’re paying attention to it more and you’re even dissecting the story a little bit more because you know that there are lessons to be told.” [Pilot FG]
	**“**I thought it was really informative, like in an interesting way. It wasn’t just someone sitting there saying: take your kids to the dentist twice a year or use fluoride. Like, I thought it was nice. Interesting.” [Community 2, FG2]
	“Well that’s the good thing about stories, ’cause they, you know, they can just relate to it, it changes personalities and it helps them remember.” [CAB meeting]
Relevance of oral tradition in light of digital and modern communications	**“**Well, … a couple visuals, at least a board or just something. ’Cause the story — you know, I think it was like, what, 15? 10 or 15 minutes long. And maybe when — I don’t know, just something to look at or something while he’s talking.” [Pilot FG]
	**“**And if you guys are going to do like individual talking, it would be like interesting for kids to see like puppets and stuff like that or — that would make it more interesting for them.” [Community 1, FG1]
	“And, but I think is what we don’t do that, and I think we’re used to, you know, like TV messages get, you know, get, you know, sound bites. And so I think we don’t really process traditional stories in the way we did at one time, just because of the other — all the other stimuli that we have in our lives.” [KI–03]
**Critiques of characters and messages**	“A lot of the parts were good. I mean, the beaver and then with the kids and then with the rabbit, you know, it was all why those characters were in the story and they all had a different message.” [Community 3, FG1]
Dissonance between modern concepts and the traditional storytelling format	“There were a few things that just kind of gave me pause with regard to the — being a storyteller, you kind of want to stay within the era and when you jump out of the era and you put things in that are current day, it kind of pulls you out of the story.” [KI–01]
	“[S]o I would really stress that this is a contemporary story in today’s world but told in the old way, or something like that. Because the kids will say, ‘Oh, they didn’t have fluoride,’ you know. Kids are pretty smart [chuckles], and they would pick up on something like that, I would think. ‘What? They didn’t have fluoride back then [chuckles]!’ But, just, you know, I would definitely say, ‘This is a contemporary story.’ [KI–02]
	“I don’t believe that we brushed our teeth traditionally, like way back in the day, beginning of time, I don’t believe we brushed our teeth. We didn’t have to. We didn’t eat foods that caused damage to ourselves.” [Community 1, FG 1]
Concerns over bad behavior by adult characters	**“**So rather than saying, ‘Auntie gave him a pacifier,’ auntie wouldn’t have given him a pacifier.” [KI–01]
	**“**Maybe he [Coyote] should be a kid, yeah, a 12-year-old maybe, not like a grown man, giving kids tainted candy.” [Pilot FG]
	“Um, it says, um, coyote’s mother, um, didn’t know, but she was responsible for coyote’s pain. Is that — do you guys feel like that’s ok, or is that a little bit too harsh? Is that making this person, that we want to make the parents — what I tried to do is make the parents look very strong, and you know very able to do these things, but was that making coyote’s mother look — did it put her in a bad light? Is that something that needs to change? Or, was this appropriate for the rest of the story?” [CAB Meeting]
Adaptation based on local audiences and tribes	“Because each tribe usually starts their stores in a certain way, and so it just depends on where the story’s being told as to how you would start it.” [KI−02]
	“For every tribe has — that I’ve worked with — has some type of trickster, you know, a figure that’s both a kind of a clown and a teacher at the same time.” [KI–03]
**Critiques of health messaging and targeting**	“I liked the thought that went into it because it shows — to me, if you start telling this story to young mothers and pregnant moms, it kind of brings up the importance that we don’t all know about.” [KI - 02]
Moving away from shame and blame	“I think that, often stories are a gentler way of doing health education and not as confrontive [sic].” [KI–03]
	“And so, you know, so that if there’s no judgment about how we parent, you know, we’re more likely to be defensive. And so I think stories do that work very well.” [KI–03]
Need for messages to relate to diverse households	“Maybe adding male characters into it because it’s primarily a female character base using Coyote. Adding some grandparent characters. Auntie. You already have an auntie but maybe the uncle character, adding those kinds of characters into it so that everybody who hears it can relate something to them, ‘Oh, this is me.’ That’s what a story’s all about, ‘This is me.’” [KI–01]
	“And I mean, there may be kids out there who are just being raised by dads or kids out there that are just being raised by moms.” [Community 1, FG2]

Abbreviations: CAB, community advisory board; FG, focus group; KI, key informant.

a For quotations selected from focus groups, the community in which the focus group was conducted is also identified, as well as which of the 3 focus groups conducted in that community produced the quote (eg, Community 1, FG 2). For quotations selected from key informant interviews, a number identifies which of the 4 KI interviews produced the quote.

Nearly all participants responded positively to the story and found it valuable to the community. This perceived value lay not only in the provision of oral health education in a format that was acceptable and accessible to the community — storytelling — but also in the story’s ability to address a knowledge gap that participants recognized as important. Though some participants reported having “heard [the information] from several different health providers, but [the information] is put in a way that’s more relatable.”

Despite the overall reported satisfaction, several participants felt that the story was too long and that the storyteller lost the audience’s attention. Additionally, there was perceived dissonance, particularly among key informant storytellers, between trying to talk about modern concepts (eg, visiting a dentist) in the setting of a traditional story. Storytellers also expressed concern with the alternating time frames of the story and the jarring nature of switching between past and present elements. One solution offered was to explain at the beginning of the story that this is a traditional story with modern elements. For example, “the pacifier would [be] something like ‘suck stick’ . . . and then qualify it by saying ‘similar to the modern day pacifier.’”

Some participants questioned the relevance of using the oral tradition to deliver a message, given the various ways in which messages and entertainment are delivered today. “[Storytelling] was done at home in the wintertime, when you were sitting around the fire trying to keep warm and you had to entertain because we didn’t have TV and all that other stuff.” Additionally, many cultural traditions are no longer daily practices in the home but instead are celebrated at infrequent events “once a year, or maybe twice a year” where “it’s always about the regalia or the dancing.” For these reasons, many participants felt that oral delivery of the story alone may limit the effectiveness and reach of message delivery and even pose a barrier to acceptance.

Participants suggested instead other means of delivery, including 1) dramatic telling of the story, with young community members playing the roles; 2) puppets; or 3) packaging the story into a book, not just for children, but for caregivers as well, to aid in their ability to remember and retell the story.

Various story characters were critiqued. Generally, characters were well-accepted, and their actions and motivations were understood, although concerns arose over portrayals of bad behavior or poor decision making by adults in the story. For example, Coyote gives candy to children and Auntie gives Coyote sugar. Focus group participants also said that there were too many characters, adding to the story’s length, perhaps unnecessarily.

Storytellers noted the need to adapt elements of the story for different audiences and communities, emphasizing the importance of using local storytellers: “[O]n my [Tribal reference removed], Coyote is very much seen as the trickster . . . but going to [location removed], Raven is the trickster.”

Acceptance of the 10 messages of the American Academy of Pediatric Dentists about oral health delivered in the story (Table 3) appeared to be influenced by the existing oral health knowledge of participants, their past experiences with dental health and services, and their ability to act on the messages provided in the story. For example, participants found some story elements confusing, such as how cavities could be spread like germs: “I didn’t know cavities jumped like lice.” Participants reported that they had received conflicting advice from others on oral health care, including from dental professionals. Some voiced confusion as to when to start taking a child to the dentist, the application and timing of fluoride varnish, and whether fluoride toothpaste is safe for use on children. Specifically, they “didn’t hear it telling how many times to brush your teeth or how many times to brush your kid’s teeth.” As a result, participants requested clarification on oral health topics such as flossing, mouthwash, orthodontic care, tobacco products, and the long-term consequences of delaying dental care.

**Table 3:** Perceived Barriers to Accepting and Acting on Ten American Academy of Pediatric Dentists Guidelines, Storytelling Intervention Addressing Oral Health Among American Indian and Alaska Native Women, Northern California, September 2012–February 2014

Guideline	Selected Participant Remarks of Perceived Barriers^a^
**1. Visit the dentist 2 times per year**	“And that’s a big thing, that’s a big distance between [location removed] and [here] . . . We have the medical [clinic] there, but there’s no dental. So there you get an overload of people going all the way to [location removed], where you have to wait a whole month, and then if you miss an appointment then that’s two more months if you miss, and if not you have to go all the way to [location removed], which is about the same distance. If they have that clinic over here, that would take a lot of, load off appointments, and you wouldn’t have to drive so far.” [CAB member]
**2. Fluoride varnish applications 2 to 4 times per year**	“I know when I take my kids, sometimes they [dental staff] say, ‘Oh, maybe we’ll put the varnish on their teeth,’ but they don’t make it sound like it’s — like they’re able to get it done and they’re able to get it twice a year and how important it is when they’re really young, you know?” [Pilot FG]
**3. First dental visit by age 1**	“Well, I don’t think . . . even you didn’t know that. You know, gee, you should take your kids . . . Like even me as a new grandma and my kids are all grown, so as a grandma with new babies it’s all new to me. I’m like okay, well, she only has a few teeth. I didn’t know I should’ve took her to the dentist already to have a checkup. So, you know, this is all new for me too because, yeah, I didn’t know.” [Community 1, FG 2]
**4. Be aware of bacteria transfer**	“I don’t understand how if you eat something and — like if I wipe her bottle off, how is she going to get a cavity from just that one? I didn't know cavities jumped like lice. Like that’s the visual it gave me, like it’s jumping around. I’m like what? How does that happen?” [Pilot FG]
**5. Adults should brush their own teeth daily**	“[Name of water source], you know it was all junky-looking? You don’t want to be brushing your teeth with that stuff.” [Community 2, FG3]
**6. Wipe baby’s gums or brush your child’s teeth daily**	“I liked how they actually talked about brushing the teeth or about the fluoride toothpaste or about wiping the gums, like I liked that. They made it relatable to today’s world.” [Community 2, FG2]
**7. Use fluoridated toothpaste**	“I was always told fluoride was not good for them to use because kids like to eat their toothpaste and swallow it, so it’s not supposed to be good for them. So I don’t even know for sure if I’m using fluoride toothpaste.” [Community 1, FG1]
**8. Reduce sugary drinks**	“I’m really glad you have the juice in there [laughter]. My grandma thinks I’m a child abuser because I don’t give my kids juice. I’m depriving them of very important nutrition.” [CAB Member]
**9. Reduce sugary foods**	“Even if you live in [location removed], if you’re on a limited budget, of course the overly processed foods tend to be a lot cheaper, and the markets in the poorer areas of town tend to hike up their prices as well, so they’ll go for the bulk highly processed food.” [CAB Member]
**10. Only water before bed time**	“I was going to say it seems like a pacifier and the milk before bed . . . people don’t think about that, because my mom did that to me, her mom did that to her, so on and so forth. So, it’s one of those things that no one thinks about because everyone’s been doing them forever.” [Pilot FG]

Abbreviations: CAB, community advisory board; FG, focus group member.

a For quotations selected from focus groups, the community in which the focus group was conducted is also identified, as well as which of the 3 focus groups conducted in that community produced the quote (eg, Community 1, FG 2). For quotations selected from key informant interviews, a number identifies which of the 4 key informant interviews produced the quote.

Participants expressed dissatisfaction with the ability to act on the oral health messages in the story because of a lack of oral health support, education, and oral health care services in the community (Table 3). Other barriers, such as availability of community-based oral health professionals and education, reliance on emergency oral health care, inability to afford oral health supplies, poor access to healthy foods, and the absence of clean or fluoridated drinking water were mentioned. Nearly unanimous concern was expressed about the unrealistic tenor of messages related to child nutrition when communities suffer from food insecurity and hunger and schools do not provide foods with high nutritional value. Additionally, messages discouraging food or toothbrush sharing, for example, seemed in conflict with existing community values — “sharing mouth-to-mouth is a big part of tradition.” However, despite concerns related to actionable messaging, participants generally felt that the story and messages were culturally appropriate, would be well-received, and were needed.

Participants liked that the messages avoided past tactics in health messaging that attempted to “shame and blame” people into better behaviors. The story messages instead came in the form of a story that was “very nonthreatening . . . Coyote gets to be the bad guy in this, rather than saying to parents, ‘you should be doing this’ and people thinking, ‘I don’t do that; does that mean I’m a bad mom’?” Interestingly, many participants did not realize that the story messages were primarily intended for adult audiences. Rather, they felt the story did and should target all community and family members, including children, recognizing that children are being raised in diverse households and that, “traditionally, things aren’t supposed to be done with partiality, but for all Indians as a whole.” Participants also noted that family dynamics, both in the present and across generations, heavily influence behavior. Thus, any messaging about oral health should recognize those dynamics and the difficulties in encouraging someone to do something that others in their households are not doing, are relaxed about, or actively oppose.

## Discussion

We developed and evaluated a story that used a traditional format for use among northern California AIAN communities to improve their knowledge, beliefs, and practices related to child oral health. The primary objective of this qualitative analysis was to evaluate this potential delivery mechanism for oral health promotion messages. Overall, participants were receptive to the story, described it as culturally appropriate, and liked the use of relatable characters. Participants considered the messages about oral health to be valuable because they moved away from the historic approach of shame and blame (20). Important concerns arose about the story itself — including delivery, content, length, and messages that conflicted with normative community values — and the intended target audience by associating the story as a learning method for children instead of adults. Key informants, primarily elders in the community, spoke of a strong historical tradition of storytelling in northern California AIAN communities; however, focus group participants’ feedback raised some doubts about the relevance and frequency of storytelling in these communities today. The influence of modern technology and digital communications (eg, smartphones, television) may weaken the acceptability of the oral tradition. In fact, the mention of other mechanisms of delivery, such as digital or pictorial, by focus group participants supports evidence that digital stories may prompt more thoughtful reflection among viewers and aid in communicating with children or communities who have low literacy levels or discomfort with writing or language (10,13,14).

The success of a storytelling intervention to improve health-related knowledge and behavior depends on the acceptability of storytelling as a delivery mechanism. Recent research demonstrated a higher level of cultural engagement by children the more they are able to recall story content (21). When tailoring a story to an AIAN community, it may be appropriate to further tailor it to the level of cultural engagement of children or adults receiving the story. Participants noted the need for community-specific adaptation during the evaluation of the storytelling demonstration. This could include adapting a story for other AIAN communities that may have differing personalities assigned to different animals (eg, Raven being a trickster instead of Coyote). This identified need reinforces findings that stories should use animals that are found in the tribe’s natural environment and the need for awareness of the different roles that animals have among various tribes (16). Another adaptation that was raised by key informants was that of changing wording when no equivalent word exists for a medical term, for example, pacifier. Participants in this study also noted the value of visual aids, family dynamics that traverse generations, and experiential learning. Tailoring to community-specific cultural values has been shown to improve educational tools and interventions provided to AIAN communities (22). Large-scale studies evaluating the effectiveness of a storytelling intervention aimed at improving uptake of knowledge and translation to behavioral change would need to balance issues of intervention fidelity and community context, especially for interventions grounded in social behavioral theories. Given that interpretations of characters, as one example, may vary slightly across communities, an intervention’s “active ingredient” (ie, the reasons why an intervention is thought to be effective), and fidelity to it, would have to be carefully considered.

Despite culturally appropriate, community-specific delivery of messages, the community context within which individuals reside may modify their capacity for behavior change, thereby limiting the potential benefit of any messaging intervention, including storytelling. In our evaluation, participants repeatedly mentioned the lack of opportunity for members of their AIAN community to act successfully upon the story’s oral health messages because of the community context. The relative absence of oral health professionals working in rural and remote locations and consequent poor access to dental services, lack of fluoridated or quality water, transportation difficulties, poor access to healthy foods, and an inability to afford supplies and services conflicts with the story’s messaging. The participants’ reactions to the story provided commentary on the system constraints they face. These references to the AIAN community-specific context uphold existing literature, which comments on the geographic isolation of AIAN tribes and the resulting barriers to help-seeking behaviors (16). Unless planned interventions aim to concomitantly address these barriers, health messaging on its own may have limited impact. Recently, there has been a favorable shift toward multilevel interventions to address disparities in health — a shift that aims to combine multiple levels of influence on health to enhance the effect of interventions (23).

Our study had limitations that point to a need for further research. Our story was presented to and evaluated by pregnant AIAN women and AIAN mothers of young children in 3 rural northern California tribal communities. The extent to which the story’s traditional elements or the educational format would be acceptable to an audience comprising men and children as well as women, or other AIAN groups or urban AIAN communities is not known. Similarly, the extent to which our convenience sample may, or may not, be representative of other AIAN mothers in northern California or elsewhere is not known. In addition, this study was not able to determine whether the overall value of the story reported by participants was primarily a function of the perceived need for the messages or primarily a function of an appreciation for the message delivery mechanism, that is, storytelling. It is possible that this population would equally value oral health messages delivered in some other way. Future research could compare participant reactions to the same messages delivered in a variety of ways (eg, media, puppetry or other dramatic renditions, video.) and by a variety of lay and professional narrators.

The effort to honor a community’s values and traditions is paramount when implementing successful health messaging interventions. However, careful attention must be made to the delivery mode, content, and targeting with initial and continual iterative feedback from community members to make these messages engaging, appropriate, relatable, and inclusive.

## Appendix Coyote and Little Man

This appendix is available for download as an Adobe PDF.
