# Supplementary material for: Using Storytelling to Address Oral Health Knowledge in American Indian and Alaska Native Communities
Source: Prev Chronic Dis. 2018 May 24;15:E63. doi: 10.5888/pcd15.170305 (PMC5985855; doi:10.5888/pcd15.170305)
Supplement: Supplementary file 1 [file 17_0305Appendix.pdf]

The key oral health messages (1-10) are listed in the right margin;
It is important that all these messages are conveyed to the listeners.

Coyote and Little Man

A long time ago, when Coyote was very young, Coyote's Auntie would take care of him from time to time. She would never do anything to hurt him, and cared for him like her own. One afternoon Coyote was fussy, and so his auntie gave him a pacifier to calm him. As he played, the pacifier fell out of his mouth and onto the dirt. His auntie looked at Coyote, "Don't cry Coyote, I will clean it for you." **She stuck the pacifier into her own mouth to get all of the dirt off and then put it back into Coyote's mouth.** It was on that very day that Little Man left the Auntie's mouth and traveled into Coyote's mouth on his pacifier. **Auntie also used to lay him down to sleep with a bottle of milk or juice. Little did she know that those drinks pooled in Coyote's mouth as he slept and the sugar** helped Little Man to grow stronger and hurt little Coyote's teeth.

> 4. Bacteria transfer
>
> 10. Before bed, only water

When Coyote grew older, he **ate soooo much sugar.** The elders often told Coyote to **eat less sugary foods and drinks,** but he didn't listen. And that Little Man in there, he loved the sweets too. Whenever Coyote ate, he fed that Little Man and all of his family, and the more sugar they ate, the more destructive they got to Coyote's teeth too. Even **as an adult, the elders told Coyote to brush his teeth daily**, but he never listened... And now Coyote was in so much pain, he just wasn't himself.

> 8. Drink less sugar
>
> 9. Eat less sugar
>
> 5. Adults should brush their own teeth daily

Coyote began to howl. Coyote was very uncomfortable that day and he didn't know why. As he walked along he began to talk to that Little Man

living inside his mouth. He lived there with his large family, but he was the only one who talked to Coyote. "Little Man in my mouth, how are you today?" asked Coyote. "I am well." "Do you know about the pain I am feeling today?" asked Coyote. "No," said Little Man. "Come on," said Coyote, "you must know something, it is right there where you are."

Coyote continued walking along with pain in his mouth and howled again. "Little Man in my mouth, are you sure that you don't know why my mouth is in pain?" "You know what Coyote? You have been good to us, you feed us well with candy and soda and you never brush your teeth, but my family has grown and there are more of us now. We need to find new places to live. Perhaps this crowded space is the reason for the pain in your mouth."

Coyote continued thinking about how he'd get Little Man's family out of his mouth so they wouldn't be so crowded, and just then Little Man saw Beaver and thought of a plan. "**Coyote, take a piece of gum, as you chew it, my family will jump onto that piece of gum**. Then go on to Beaver, he will see the gum and he will want a piece." Coyote approached Beaver, "Hello there Beaver," while smacking on his gum. "Hello Coyote, what are chewing on, it sure does look good!" "It is the best gum ever, would you like a piece?" Coyote asked. "Yes please." Coyote turned and acted as if he was getting another piece of gum, but what he really did was take that piece of gum out of his mouth and then he carefully placed it back in its little silver wrapper. Beaver took the gum and without looking popped it into his mouth. As soon as he put it in his mouth he realized what Coyote had done, and he spit the gum to the ground. "What kind of trick is that? **You should never eat food that someone else has already chewed on. I won't even chew food for my small children, you can pass harmful germs that way**!" Beaver scolded Coyote. Coyote felt that he had done the job, but Beaver grabbed his toothbrush that he always carried and began **brushing his beaver**

4. Bacteria transfer

4. Bacteria transfer

5. Adults should brush their own teeth daily

teeth. Coyote could tell that Beaver was very angry but he was happy he had done his job. But Coyote still felt the pain in his mouth, he howled, and he began to talk to Little Man again.

“I have done what you asked Little Man but my mouth still feels pain.” “You have failed me Coyote. That was a good plan, and many of my family were on that gum and made it to Beaver’s teeth, but when Beaver brushed his teeth, he brushed them all away.” Coyote was discouraged, “I didn’t know that. I’m still in so much pain, I’ll do anything, tell me, what can I do now? I promise, I’ll do better next time.” “There are still too many of us in your mouth, you need to find us another home. You will not be able to trick an adult to do this. You will have to trick a young one, they will not be suspicious.” “Ok, I will just be helping your family to find a new home.”

Just then, Bear and her little ones came down the trail walking toward Coyote. “There are Bear’s cubs, let’s stop them. Do you have candy Coyote?” asked Little Man. “Of course I have candy, that’s all I ever eat!” said Coyote. “Okay now, take that candy and rub it all over your teeth, my family will hop onto the piece of candy. When the cubs get here, give them that candy, children love candy, they will take it,” said Little Man. “Ok,” said Coyote, “I will do it.” But, the sugar from the candy made his mouth hurt more. “Hello Coyote, why are you making that funny face?” Bear asked. “Me? Uh, I’m just thinking real hard. That’s the way I look when I’m thinking. Anyway, how are you and your little ones?” “We are well, we just saw Hawk, the dentist, and she said my cubs’ teeth are good.” “The dentist? Why would you take them to the dentist? Aren’t your little ones too young?“ asked Coyote. “Coyote! **You’re supposed to go to the dentist twice a year,**” said Bear. “But your littlest one doesn’t even have teeth,” said Coyote. “Silly Coyote! My littlest one’s first birthday is next week, it’s **important to take them to see the dentist before they turn one** even if they don’t have teeth yet. Oh, and I want to make sure they get **fluoride varnish** too.” Coyote

1. Take your kids to the dentist 2x year

3. See dentist by age 1

2. Fluoride varnish 2-4x a year

was so confused, so he asked, “Flouride varnish? Is that for your teeth? Bear said, “**Yeah Coyote, if they get it 2-4 times a year, it helps protect their teeth.**” Coyote wasn’t really listening because he was thinking about the pain in his mouth and giving the cubs that candy. “Give them the candy Coyote,” Little Man whispered. Coyote turned to the cubs, “And how are your little cubs doing today? How about some candy?” Bear was furious, “What! What kind of trick are you pulling Coyote? **Too much candy can be bad for your teeth!”** Coyote didn’t know why Bear was upset. Little Man whispered to Coyote, “Don’t give up Coyote, take a bite of your apple and offer them that.” Coyote turned to Bear, “Bear, I didn’t know too much sugary foods is bad for teeth.” He grabbed an apple and took a big bite, “Now little cubs, surely you would like to try this apple. I will share it with you.” Bear could see Coyote was trying to be nice, “**No thank you Coyote. When you share food, you share germs.”** “Come now little ones,” Bear said and they walked away from Coyote.

2. Fluoride varnish 2-4x a year

9. Eat less sugary foods

4. Bacteria transfer

“What is Bear even talking about?” Coyote asked Little Man. “I have failed again.” Coyote howled again, his mouth was still in pain. It was getting dark now and most of the young ones were getting into bed, they had to act fast and come up with a new plan.

It was then that they came across the house of Rabbit. “There are two little ones in there, but how will I get in there?” Coyote said to Little Man. Coyote walked along and thought about this. He was walking along and saw a berry bush when Little Man gave him a plan. “Those children love juice, take these berries and squeeze the juice out of them. When you have a cup of juice, take it to the little ones.” Coyote liked the idea. He squeezed the berries into a large cup of juice. “**Now take a drink of this juice and when you do, my family will grab onto the cup, then when someone takes a drink my family will have a new place to live**.” “Ok,” Coyote agreed to the plan. As he took a drink of the juice his mouth hurt even more. He took the cup, walked to the

4. Bacteria transfer

Rabbit door and knocked, holding the cup of juice behind his back. Rabbit opened the door and welcomed Coyote into his house. “Hello Coyote, how are you this evening?” “I am well Rabbit, I just came to visit your family and look in on those two little ones. I have a story to tell them if it’s ok.” “Sure, that would be ok; they are ready for bed, they have brushed their teeth for the day and are all tucked in. It’s perfect timing for a bedtime story, Coyote.” So Coyote did, he went back and told those little ones an exciting story. When he finished, he told the little rabbits that he had a fresh cup of juice, just for them. They smiled and Coyote noticed that the youngest one had no teeth. “This will not work for the youngest one, he has no teeth,” Coyote whispered to Little Man.“Yes it will still work. My family can hang out on the gums and wait for the teeth to come.” The first little one took a nice drink of the juice, and then the youngest one drank. Some of the Little Man’s family made it into the mouths of the little rabbits. “Now these two will sleep, no chance of my family being washed away,” said Little Man. Just then Momma Rabbit heard the excitement of her kids and she came into the room to see what was going on. She saw the juice cup and got very upset. “What kind of trick is this Coyote?” She scooped up her littlest one. **She called for Father Rabbit to come and help the older one brush his teeth with fluoride toothpaste again.** She took a clean cloth and wiped the gums of her baby just as she had done earlier that night when she was getting them ready for bed. “**But why did you wipe the baby’s gums? He doesn’t even have teeth.” said Coyote. Mamma Rabbit sighed, “Coyote you should brush your teeth everyday and wipe the gums of young ones even before they have teeth. I also don’t let my little ones go to sleep with bottles of juice or milk; it can really hurt their teeth**.” Coyote could see that they were upset so he made his way to the door.

6. Brush kids teeth, wipe gums of baby daily

7. Fluoride toothpaste

10. Only water before bed time

Coyote was confused about why everyone was so mad at him, and he was also sad that he had failed once again, but all that he could really focus on was that pain in his mouth. Coyote howled. His mouth hurt

more now than ever; the gum, the candy and the juice were too sweet, when the sugar was on his teeth, his whole mouth hurt. Again that Little Man was silent. Coyote did not like that the Little Man in his mouth was not helping. Every step he took he grew more and more mad. And every step he took he was in more and more pain. He was thinking to himself now, so that the Little Man could not hear him, “I am a trickster, but this Little Man, he is more of a trickster than I am, everything he has asked me to do has been more harmful than good, and even my friends are upset with me for what I have done.” Coyote had walked down the path for some time, he was thinking about his day and everything that had happened when he came to the end of the road. That’s when he found a sign that read “Hawk, Community Dentist.” Now that Little Man in Coyote’s mouth, he knew who Hawk was and he started to stir. “What are you doing?” he asked. “Shouldn’t we be finding a new mouth for my family?” Now Coyote said nothing. “Coyote lets go get a soda, aren’t we thirsty?” Little Man asked, “what about some cake, or a brownie, or ice cream, you love ice cream. You are hungry aren’t you Coyote?” For the first time in a long time he didn’t want sweets or even a soda. Coyote ignored Little Man who tried to convince him to turn away from Hawk’s office. Still Coyote was silent.

When he reached the door he was met by Hawk, who was leaving for the night. “How are you Coyote?” Hawk asked. “Not Good Hawk,” replied Coyote. Coyote told Hawk about the pain in his mouth, and about the gum, the candy and the juice. As he talked the pain in his mouth grew stronger. “Hawk, even though Little Man’s family left my mouth I am still in pain. I don’t think that helped at all,” said Coyote. “Coyote you have been tricked by Little Man. You have not treated your teeth very well. Little Man and his family have done a lot of damage to your teeth. **You know if you brush your teeth every day, you can brush away Little Man and his family. They are causing you this pain.** They can cause damage to your teeth even if you don’t feel

5. Adults should brush their own teeth daily

the pain, **they can also be harmful to others' if they move from your mouth to theirs. Especially when you have little ones, Coyote, it is really important that you brush your own teeth to keep them clean.**" said Hawk. It was finally making sense to Coyote. Coyote was ashamed, he had never been tricked in such a way. Hawk cleaned and worked on Coyote's teeth, until there was no pain in his mouth. That Little Man and his family who lived on Coyote's teeth were gone. As Coyote was leaving, he turned to Hawk, "Hawk, thank you for helping me. I have learned a lot today. I feel so foolish that I have been tricked by Little Man into trying to spread harmful germs to my friends." The next day, he was back to himself since he was no longer in pain. He wanted to share what he learned with his friends. When he saw bear and her family he went to apologize to them and thanked her for teaching him about children's teeth. "**When I have little ones of my own I will bring them to the dentist before their first birthday, and twice a year after that. I will make sure they brush their teeth daily with fluoride toothpaste. And thank you for teaching me about fluoride varnish, I didn't know about that. I will make sure I get fluoride varnish put on their teeth 2-4 times a year to protect them. Oh and don't worry, I won't be sharing my food anymore because I don't want to share my germs. I also learned not to share anything that's already been in my mouth.** Later that day, he ran into Rabbit and Beaver. After apologizing to them, he shared with them more of the things that Hawk had told him about **I learned that I shouldn't eat so much sugary foods or drinks, I am going to cut back on those." And now I understand why you don't give your babies milk or sugary drinks just before bed, only water." And now I also know why you wipe your babies' gums.** After hearing this, each of his friends saw that Coyote had learned a lesson. Even Beaver.

Coyote continued to walk down a new path and thought about his future children. Even today, we still hear Coyote howling, he is

4. Bacteria transfer

5. Adults should brush their own teeth daily

1. Dentist 2x year
2. Fluoride varnish 2-4x a year
3. First dental visit by age one
4. Bacteria transfer
5. Adults should brush their own teeth daily
6. Wipe baby's gums or brush your child's teeth daily
7. Fluoride toothpaste
8. Less sugary drinks
9. Less sugary foods
10. Only water before bed time

reminding us of the pain he once felt in his mouth when he didn’t take care of his teeth.
